# Our Home on the Hillside

**Published:** 1875-01

**Authors:** 


### OUR HOME ON THE HILLSIDE.

At the head of a branch of the beautiful Genesee Valley, in the town of Dansville, in Livingston County, New York, is situated a widely-known curative establishment, bearing the above name. For seventeen years the good work of this institution has been going on. Its founder was Dr. James C. Jackson, one of the earliest and most prominent pioneers in the system of medical treatment known as "Water-Cure." As early as 1848 he adopted this system, to the exclusion of all drugs, in his establishment at Glen Haven, Cayuga County. His success here induced him to seek increased facilities and a wider field. His choice fell upon the spot which we have indicated, and it is fair to say that the selection was an admirable one. Viewed in a sanitary light, the location could scarcely be improved upon. The beautiful valley, watered by the Canaseraga River, is surrounded by mountains averaging about 1,000 feet in height, and covered with forest trees, and finely cultivated farms. The "Home," with its numerous cottages and other outbuildings, stands on the hill-side at an elevation above the valley of some 140 feet. The air is pure and invigorating, and the surroundings are of the most healthful character. The water supply for the establishment is brought from the hill-side through pipes. It comes from a spring which rises out of a slate formation, at the rate of 2,500 barrels each day. It is pure and soft and abundant.

Dr. Jackson is still at the head of the institution, but he has numerous assistants now, as the establishment is one of great magnitude. It has accommodations for not less than 250 patients, and is open from one year's end to the other. It has a reputation for the successful treatment of disease, which secures for it a large family of health-seekers at all times. Perhaps no hospital or curative institution anywhere, can show a larger per centage of cures than this. The worn-out and broken-down bodies flock to it from afar—a never ending procession,—and almost all testify that they experience relief, while nine out of every ten confess to permanent benefit.

It is not water alone that does all this, for not even the soft, cool and delicious water of that mountain spring at Dansville, however applied, is adequate to accomplish cures in this proportion. The poor bodies are certain to be badly shattered before they cease to rely upon local means for relief. So a great majority of all the sick people who apply at the Home have failed to find a healing balm elsewhere. For any single remedial agency to be competent to heal and renovate so remarkable a proportion of broken lives, would involve the discovery of a specific which would convert disease and pain into unnecessary intruders. No waters since the best days of the Pool of Siloam have proved adequate to this end.

The good results are wrought, so patients tell us, by a combination of wisely-selected forces. The social enfluences are ever on the alert. The mind is engaged, the feelings are inlisted ; the whole nature is broadened and expanded. No sufferer is allowed to feel that he is a stranger, lacking sympathy and acquaintance. The home feeling is awakened. The tired heart and brain and body are taught how to rest and be strengthened. Appropriate diet, much out-of-door life in the pure, invigorating air of the mountains, delightful social influences, and freedom from the ordinary fashionable restraints—all these supplement such a wise employment of the water treatment as long experience and a well-kept record of results prove expedient.

A great change has come over the minds of medical men with reference to the use of water in disease since the HOME was established. To use water in febrile affec.ions is far more orthodox now than to employ the lancet. The physician who would neglect to reduce abnormal temperature by applications of water would be deemed a trifle behind the age. The surgeon who would banish water-dressings would sacrifice a most important adjunct in the treatment of dislocations, fractures, and amputations. Water is no longer the bugbear of the faculty—it is a wonderful controller of diseased action. The "water-cure"—by which we mean the advantages to be derived from its imperfect and extemporaneous use—is employed by medical men every where. Of course it cannot be as well applied anywhere as at a place constructed expressly for the purpose. Knowing this, it is common for practitioners of all schools to send patients to the HOME at Dansville. Indeed, many doctors, enfeebled by disease and overwork, have placed themselves under the care of Dr. Jackson, and have abandoned medicine for a season, to seek and find health by right living in the genial atmosphere of "OUR HOME ON THE HILLSIDE

On our table is a magazine filled with good things relating to health and sanitary science, entitled *The Laws of Life and Journal of Health.* It is published monthly at the institution of which we have spoken. Its subscription is $1.50 a year. We cordially commend its teachings to all who value health, and would know the best means of retaining and restoring it.

FUEL BURNING WITHOUT SMOKE.—A fuel burning without smoke, needing no attention after lighting, and said to be especially adapted to heating railway cars, has been patented in England. It consists of a mixture of pulverized charcoal or coal with some material affording oxygen when heated, as nitrate or chlorate of potash, &c. Some cementing substance such as gum, starch or water-glass is employed to form it into cakes, which are compressed and dried at a gentle heat. Special apparatus has been devised for its combustion. The heat generated by it is most intense, and the length of time which the fire burns without replenishing, is marvellous. Here is a hint for some one who has access to plenty of coal dust of any kind. We have seen a pile of finely powdered anthracite mixed with say ten per cent. of common clay, and the whole wet up with a solution of soda and shaped into bricks, and dried, and hardly expect to see better fuel. It gives off great heat, is very durable, and kindles quite as easily as hard coal.

---

MEDICATION BY ABSORPTION.—Dr. Cooper's Pads and Belts, for the cure of rheumatic and other ills, the merits of which we have heretofore discussed at some length, appear to be accomplishing a highly useful work. They differ from the thousand and one "chest protectors" generally for sale, inasmuch as they contain healing drugs between the flannel and buckskin layers. We hear the article very earnestly commended by those who have used it. Messrs. Hegeman, the well-known druggists, are the New York agents, and the goods may be found at their several stores.

---

Keep the body moving when you are in the open air in cold weather.

